# Diverse Landscape of Group 1 Innate Lymphoid Cells Predicts the Prognosis in Patients with Head and Neck Squamous Cell Carcinoma

**DOI:** 10.3390/cancers17122047

**Published:** 2025-06-19

**Authors:** Hideyuki Takahashi, Toshiyuki Matsuyama, Hiroe Tada, Hiroyuki Hagiwara, Miho Uchida, Kazuaki Chikamatsu

**Affiliations:** Department of Otolaryngology-Head and Neck Surgery, Gunma University Graduate School of Medicine, Showa-Machi 3-39-22, Maebashi 371-8511, Gunma, Japantikamatu@gunma-u.ac.jp (K.C.)

**Keywords:** innate lymphoid cells, group 1 ILCs, NK cells, single-cell RNA sequencing, head and neck squamous cell carcinoma

## Abstract

**Objectives:** Innate lymphoid cells (ILCs) and natural killer (NK) cells represent a diverse group of innate immune populations that modulate immune responses and tissue equilibrium across various diseases, including cancer. In the present study, we analyzed single-cell RNA sequencing (scRNA-seq) data to explore the landscape and functional status of ILC subsets in patients with head and neck squamous cell carcinoma (HNSCC). **Methods**: The GSE164690 dataset, which includes preprocessed scRNA-seq and clinical data, was acquired from the Gene Expression Omnibus database. The Cancer Genome Atlas database was used to develop the survival prediction model. **Results**: A total of 95,809 immune cells were clustered into 16 immune cell clusters, among which 7278 NK cells were further subdivided into 11 clusters. Among the 11 clusters, eight NK cell clusters, two intraepithelial ILC1 (ieILC1) clusters, and one ieILC1–NK-intermediate (ieILC1-NK-int) cluster were identified. Among the ieILC1/NK clusters, ieILC1-1 exhibited the highest immunological activity and was mainly derived from human papillomavirus-positive samples. Further, ieILC1s showed higher enrichment of pathways related to inflammation and effector functions—such as inflammatory response, interferon-gamma response, and interferon-alpha response—compared to the other clusters. Moreover, we developed prognostic prediction models based on differentially expressed genes in the ieILC1/NK clusters. Risk scores of the ieILC1-1, ieILC1-NK-int, and NK clusters were identified as independent prognostic factors for shorter overall survival (OS) and progression-free survival (PFS). Recursive partitioning revealed that combining ieILC1-1 and the NK clusters strongly predicted shorter OS and PFS. **Conclusions**: Our findings highlight the diverse landscape and prognostic significance of ieILC1/NK cells in patients with HNSCC.

## Simple Summary

Innate lymphoid cells (ILCs) and natural killer (NK) cells are a heterogeneous family of innate immune cells exhibiting not only antitumoral but also protumoral activities. The objective of this study was to determine the landscape and prognostic significance of ILC/NK cells in patients with head and neck squamous cell carcinoma (HNSCC). Publicly available single-cell RNA sequencing data were analyzed, and four group 1 ILC clusters were identified: intraepithelial ILC1 (ieILC1)-1, ieILC1-2, ieILC1–NK-intermediate, and NK cells. Among the ieILC1/NK clusters, ieILC1-1 was the most immunologically active phenotype, mainly comprising human papillomavirus-positive samples. Risk scores calculated based on the differentially expressed genes of the ieILC1/NK clusters strongly predicted a poor prognosis. Our results suggest that further exploring group 1 ILCs could provide new insights into the development of cancer immunotherapy and biomarkers for patients with HNSCC.

## 1. Introduction

Innate lymphoid cells (ILCs) and natural killer (NK) cells comprise a diverse group of innate immune cells. They control inflammation, immune tolerance, and tissue equilibrium across various diseases, including cancer [1,2,3]. Unlike adaptive lymphocytes, ILCs lack antigen-specific receptors and develop independently of recombination-activating genes. Their effector functions are mediated through cytokine production in response to STAT-activating signals and interleukin (IL)-1 family alarmins [4,5]. Based on transcription factors and cytokine signatures that resemble those of T helper (Th) subsets, ILCs are classified into five subsets: NK cells, ILC1, ILC2, ILC3, and lymphoid tissue inducer (LTi) cells [1,3,6]. NK cells and ILC1s, both belonging to group 1 ILCs, express transcription factor T-bet and produce interferon (IFN)-γ, resembling Th1 cells [1,3]. ILC2s, also known as group 2 ILCs, express transcription factor GATA-3 and secrete IL-4, IL-5, and IL-13 in parallel with Th2 cells [1,3]. Group 3 ILCs, including ILC3s and LTi cells, express transcription factor RORγt and secrete IL-17, IL-22, and lymphotoxin, resembling Th3 cells [1,3].

Among the ILC subsets, group 1 ILCs, which include ILC1s and NK cells, share several key characteristics such as T-bet expression, IFN-γ production, and development dependent on IL-15 [7,8]. NK cells also express transcription factor Eomes, which distinguishes NK cells from ILC1s [7,8]. In humans, NK cells are divided into conventional NK (cNK) and tissue-resident NK (trNK) cells [9,10]; cNK cells circulate in peripheral blood and resemble CD56dim, while trNK cells are CD56bright and express tissue residency markers, such as integrin α1 (*ITGA1*, CD49a) and integrin αE (*ITGAE*, CD103) [9,10]. Among cNK cells, CD57+ cells are defined as a functionally distinct population that is highly mature and terminally differentiated [11]. ILC1s also comprise heterogeneous populations. Intraepithelial ILC1 (ieILC1) is a tissue-resident population of ILC1. It resides in the tonsil and gut epithelium, expresses alpha E beta 7 (aeb7) integrin (CD103) and NKp44, and lacks the ILC1 marker CD127 [8].

Immunological destruction is a hallmark of cancer [12,13]. Among the various immune cells, innate immunity serves as the frontline against cancer. Moreover, it facilitates adaptive immune responses, especially antitumoral T cell responses [14,15]. However, in the tumor microenvironment (TME), ILCs play a dual role, including both tumor regression and tumor promotion. This suggests that their plasticity and functional states are regulated by cytokines produced by both tumors and other immune cell types [16,17,18]. NK cells are a major subset of ILCs exhibiting antitumoral activity by producing granzyme, IFN-γ, and perforin [19,20]. Similarly to NK cells, ILC1s exhibit effector function against cancer cells by producing IFN-γ [21]. Meanwhile, several findings suggest the presence of poorly cytotoxic and protumoral ILC1s and transforming growth factor-β may induce the conversion of NK cells into protumoral ILC1s that produce less IFN-γ [22,23,24]. Similar to ILC1s, both the antitumoral and protumoral roles of ILC2s and ILC3s have been explored but remain unclear [25,26]. A better understanding of the plasticity and functional state of ILCs is crucial for overcoming immune destruction in various cancers, including head and neck squamous cell carcinoma (HNSCC).

In the current study, we analyzed single-cell RNA sequencing (scRNA-seq) data to examine the landscape and functional status of ILC subsets in patients with HNSCC. In addition, we constructed prognostic prediction models based on differentially expressed genes (DEGs) in ILC subsets. Our results revealed the diverse landscape and prognostic significance of group 1 ILCs in patients with HNSCC.

## 2. Materials and Methods

### 2.1. Acquisition of the GSE164690 Dataset from a Publicly Available Database

The GSE164690 dataset, including preprocessed scRNA-seq and clinical data, was acquired from the Gene Expression Omnibus database. CD45-positive cells and peripheral blood lymphocytes from 17 matched tumor and peripheral blood samples, including six from human papillomavirus (HPV)-positive and 11 from HPV-negative HNSCC patients, were analyzed using the Seurat v4 R package.

### 2.2. Processing of scRNA-Seq Data

Cells expressing fewer than 100 genes were excluded. Global-scaling normalization was performed using a scale factor of 10,000. In total, 2000 features exhibiting high cell-to-cell variation were identified for downstream analysis. Nonlinear dimensional reduction was performed using Uniform Manifold Approximation and Projection (UMAP). Unsupervised hierarchical clustering of cells was performed using the FindClusters function at a resolution of 0.2, resulting in 16 distinct clusters. DEGs in each cluster were identified using the FindAllMarkers function. The xCell R package (version 1.1.0), a gene signature-based deconvolution method, was used to evaluate the enrichment of various immune cell types in each cluster. The mean normalized counts in each cluster were used as inputs for the xCell tool.

Subsequently, 7278 NK cells were extracted and automatically clustered into 11 NK clusters using the FindClusters function at a resolution of 0.4. To characterize the 11 NK clusters, the abundance of NK subsets was estimated using CIBERSORTx (https://cibersortx.stanford.edu), a deconvolution tool that estimates the abundance of member cell types in a mixed-cell population using gene expression data. Normalized gene expression profiles of bulk-sorted human NK cells (CD56 bright, CD56dim/CD57pos, and CD56dim/CD57neg), ieILCs, and ILC3s were obtained from the GSE112813 dataset and used as reference signature matrices for CIBERSORTx. Alternatively, single-sample gene set enrichment analysis (ssGSEA) was performed to quantify the activated pathways and processes in each cluster using the escape R package (version 2.5.3).

### 2.3. Development of an ieILC1/NK-Related Prediction Model

Based on DEGs in each ieILC1/NK cluster (adjusted *p*-value < 0.01), we developed an ieILC1/NK-related prediction model for prognosis in patients with HNSCC. RNA sequencing data (Illumina Hiseq RNAseq V2, normalized counts) and clinical data of patients with HNSCC in The Cancer Genome Atlas (TCGA) database were downloaded from the FireBrowse website (http://firebrowse.org/). A total of 520 patients with HNSCC (97 HPV-positive and 423 HPV-negative patients with HNSCC) were analyzed. For primary screening, we performed univariate regression analyses of the overall survival (OS) and progression-free survival (PFS) using a Cox proportional hazards model. Genes with *p* < 0.05 were considered primary predictive features (Supplementary Table S1). Least absolute shrinkage and selection operator (LASSO) regression, a dimensional reduction method, was employed to select suitable features using R package glmnet (version 4.1.8). Tenfold cross-validation was performed to tune the optimal lambda (λ) that yields the minimum mean cross-validated error. For each sample, a risk score was calculated according to the following formula: risk score = e^Σ(coefficient × (gene expression − average (gene expression)))^. Survival curves were constructed using the Kaplan–Meier method and compared using the log-rank test. Receiver operating characteristic curves were plotted separately for survival analysis to verify the optimal cutoff value for each risk score. Multivariate regression analysis was performed for both the risk scores and the clinical variables using the Cox proportional hazards model. Variables were included in the multivariate regression analyses when *p*-values were <0.05 in univariate analyses. Subsequently, recursive partitioning analysis was performed to generate risk models using R package partykit (version 1.2.23). Independent prognostic factors in the multivariate regression analyses were included.

### 2.4. Statistical Analysis

Data analyses were performed using R (version 4.4.1; The R Foundation for Statistical Computing, Vienna, Austria) in combination with R Studio (version 2023.6.0.421; R Studio, Boston, MA, USA). Heatmaps were constructed using R package pheatmap (version 1.0.12).

## 3. Results

### 3.1. Tumor-Derived CD45+ Cells and Peripheral Blood Cells Were Clustered into 16 Immune Cell Types

**Figure 1:**
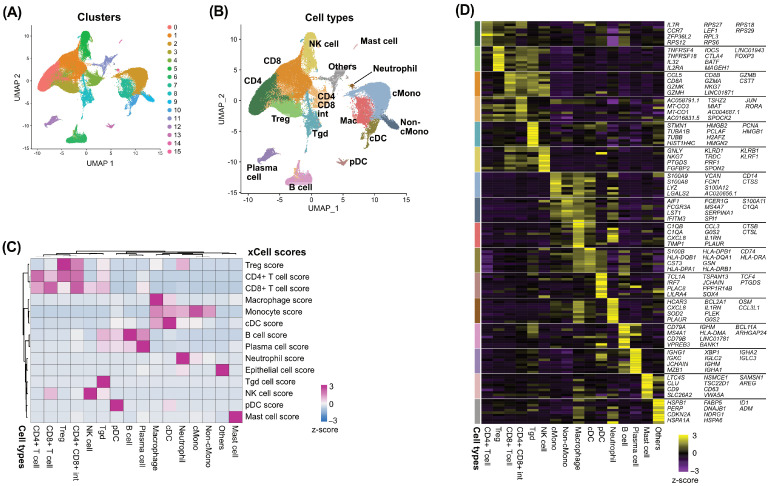
Tumor-derived CD45+ cells and peripheral blood cells were clustered into 16 cell types and scRNA-seq data of 17 pairs of CD45-positive cells and peripheral blood lymphocytes isolated from patients with HNSCC were analyzed. A total of 95,809 cells were automatically clustered into 16 clusters. (**A**) UMAP showing the distribution of 16 clusters. (**B**) UMAP showing the 16 immune cell types. (**C**) Heatmap showing the cell type enrichment scores in each cell type calculated using the xCell tool. (**D**) Heatmap showing the top DEGs in each cell type. Note: scRNA-seq, single-cell RNA sequencing; UMAP, Uniform Manifold Approximation and Projection; DEG, differentially expressed gene.

Initially, we analyzed scRNA-seq data from the GSE164690 dataset. After quality control, 95,809 cells were clustered into 16 clusters using unsupervised hierarchical clustering (Figure 1A). The immune cell type of each cluster was then defined based on the cell type enrichment scores and DEGs (Figure 1B–D, Supplementary Figure S1).

### 3.2. Sub-Clustering of the NK Clusters Indicated the Presence of ieILC1, NK, and ieILC1–NK-Intermediate Clusters

**Figure 2:**
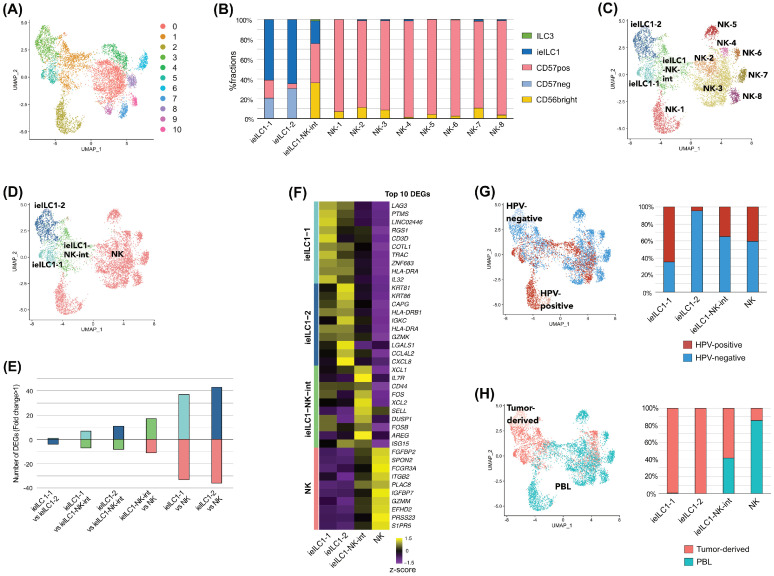
Sub-clustering of the NK clusters indicates the presence of ieILC1, NK, and ieILC1–NK-intermediate clusters. A total of 7278 NK cells were automatically sub-clustered into 11 clusters. (**A**) UMAP showing the distribution of 11 clusters. (**B**) Bar graphs showing the deconvoluted compositions of 11 clusters based on the gene expression signature of bulk-sorted human NK cells, ieILC1s, and ILC3s. (**C**) UMAP showing two ieILC1 clusters, eight NK clusters, and an ieILC1–NK-int cluster. (**D**) UMAP showing two ieILC1 clusters, a merged NK cluster, and an ieILC1–NK-int cluster. (**E**) Bar graphs showing the number of DEGs between all four ieILC1/NK clusters. (**F**) Heatmap showing the top DEGs in each ieILC1/NK cluster. (**G**) UMAP/bar graphs showing the distribution/proportion of cells based on the HPV status. (**H**) UMAP/bar graphs showing the distribution/proportion of cells based on sample types. Note: UMAP, Uniform Manifold Approximation and Projection; ieILC, intraepithelial innate lymphoid cell; DEG, differentially expressed gene; HPV, human papillomavirus; PBL, peripheral blood lymphocyte.

Next, we performed the sub-clustering of the NK clusters. In total, 7278 NK cells were clustered into 11 clusters using unsupervised hierarchical clustering (Figure 2A). To estimate the cell type of each cluster, we analyzed the fractional compositions of 11 clusters using CIBERSORTx with previously published gene expression profiles of bulk-sorted human NK cells (CD56 bright, CD56dim/CD57pos, and CD56dim/CD57neg), ieILCs, and ILC3s (Figure 2B). For each cluster, DEGs were calculated to estimate the cell types in the 11 clusters (Supplementary Figure S2A). Further, ssGSEA was performed to quantify activated pathways and processes in each cluster (Supplementary Figure S2B–C). Based on these results, we identified two ieILC1 clusters (ieILC1-1 and ieILC1-2), eight NK clusters (NK-1, NK-2, NK-3, NK-4, NK-5, NK-6, NK-7, and NK-8), and an ieILC1–NK-intermediate cluster (Figure 2C). The cell distribution and the DEG profile of the NK-1 cells were different from those of the other NK clusters. This difference might be attributable to the difference between samples (Supplementary Figure S2D). Based on similarities in the fractional compositions, the eight NK clusters were merged into a single NK cluster (Figure 2D). Based on pairwise comparisons between all four ieILC1/NK clusters, the highest number of DEGs was observed between ieILC1-2 and NK cells, whereas the lowest was detected between ieILC1-1 and ieILC1-2 (Figure 2E). DEGs in each cluster were calculated to further characterize the ieILC1/NK clusters (Figure 2F, Supplementary Figure S2E). Regarding the HPV status, most ieILC1-2 cells were from HPV-negative samples, whereas over 60% of ieILC1-1 cells were from HPV-positive samples (Figure 2G). Both ieILC1-1 and ieILC1-2 mostly comprised tumor-derived CD45-positive cells, whereas most NK cells comprised peripheral blood lymphocytes (Figure 2H).

### 3.3. The Most Immunologically Active Phenotype Among the NK Clusters Was ieILC1-1

**Figure 3:**
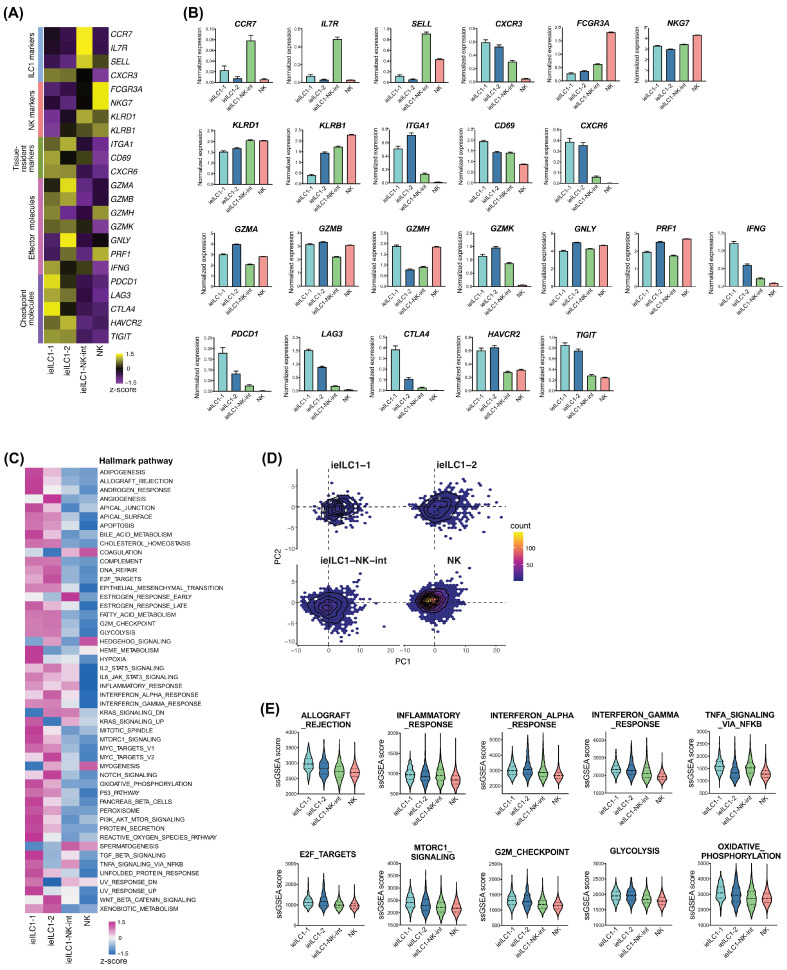
The most immunologically active phenotype among ieILC1/NK clusters is ieILC1-1. (**A**) Heatmap showing the expression of ILC1 markers, NK markers, tissue-resident markers, effector markers, and checkpoint molecules. (**B**) Bar graphs displaying the expression of the genes shown in (**A**). (**C**) Heatmap showing the ssGSEA scores in the ieILC1/NK clusters. (**D**) PCA plot based on the ssGSEA scores shown in (**C**). (**E**) Violin plots displaying the ssGSEA scores related to effector function, cell growth, and cell metabolism. Note: ieILC, intraepithelial innate lymphoid cell; ssGSEA, single-sample gene set enrichment analysis; PCA, principal component analysis.

We analyzed the gene expression of both cell type markers and effector/checkpoint molecules to further understand the ieILC1/NK clusters (Figure 3A,B). Interestingly, the ieILC1–NK-intermediate cluster exhibited the highest expression of specific ILC1 markers, including *CCR7*, *IL7R*, and *SELL*. The expression of NK markers, including *FCGR3A*, *NKG7*, *KLRD1*, and *KLRB1*, was the highest in NK cells. The expression of the tissue-resident marker *ITGA1* was the highest in ieILC1-2, whereas that of *CD69* and *CXCR6* was the highest in ieILC1-1. The expression of effector molecules differed among the ieILC1/NK clusters. Notably, the expression of checkpoint molecules, including *PDCD1*, *LAG3*, *CTLA4*, and *TIGIT*, was the highest in ieILC1-1, whereas that of *HAVCR2* was similar between ieILC1-1 and ieILC1-2.

In addition, ssGSEA was performed to quantify the activated pathways and processes (Figure 3C–E), revealing that various hallmark pathways, especially those related to effector function, cell growth, and cell metabolism, were highly upregulated in ieILC1-1.

### 3.4. Prognostic Prediction Models Constructed Using LASSO Regression and Recursive Partitioning Analysis

**Figure 4:**
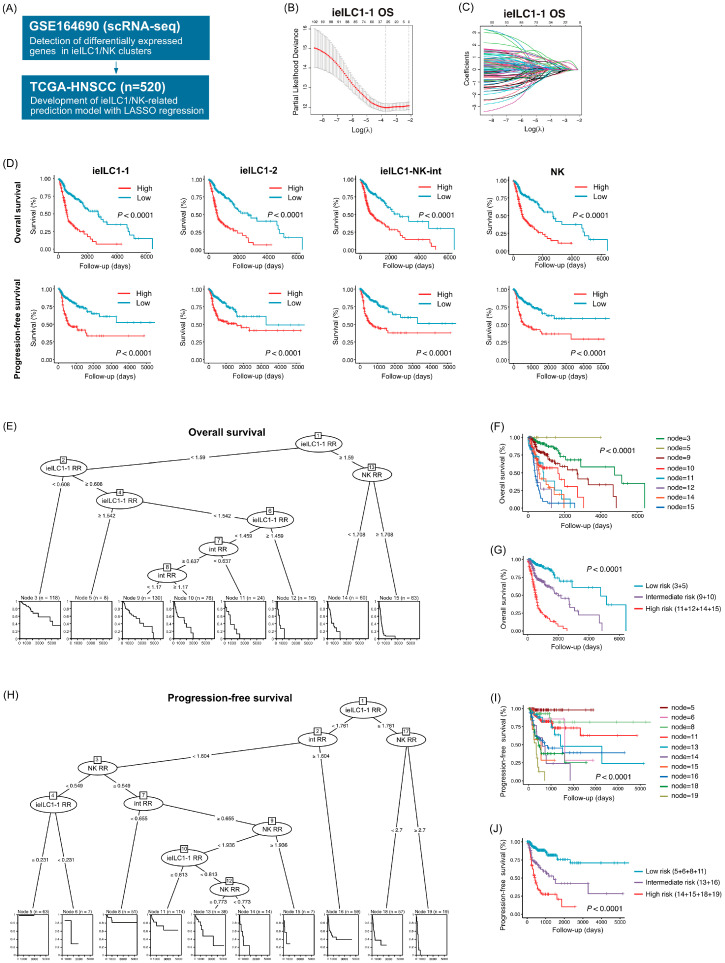
Prognostic prediction models constructed using LASSO regression and recursive partitioning analysis. (**A**) Schematic diagram illustrating the construction of prognostic prediction models. (**B**) Plot showing partial likelihood deviance versus log (lambda) in ieILC1-1 associated with the overall survival. The vertical dotted line indicates the lambda value with the minimum error and the largest lambda value for which the deviance was within one standard error of the minimum. (**C**) LASSO coefficient profiles of the DEGs in ieILC1-1 associated with the overall survival. (**D**) Kaplan–Meier survival curves based on the ieILC1/NK risk scores. (**E**) The conditional inference tree for the overall survival. (**F**) Kaplan–Meier survival curves based on the terminal nodes identified in (**E**). (**G**) Kaplan–Meier survival curves based on the merged terminal nodes stratifying patients into three risk groups. (**H**) The conditional inference tree for progression-free survival. (**I**) Kaplan–Meier survival curves based on the terminal nodes identified in (**H**). (**J**) Kaplan–Meier survival curves based on the merged terminal nodes that stratified patients into three risk groups. Note: LASSO, least absolute shrinkage and selection operator; ieILC, intraepithelial innate lymphoid cell; DEG, differentially expressed gene.

We constructed prognostic prediction models based on the DEGs of each ieILC1/NK cluster using the TCGA cohort (Figure 4A). After primary screening using univariate Cox regression analysis, we performed LASSO regression to select suitable features from among the DEGs for each ieILC1/NK cluster based on both OS and PFS. Furthermore, we calculated the risk scores for each cluster (Figure 4B,C, Supplementary Figure S3). The risk scores significantly correlated with the HPV status, primary lesion, T factor, and TNM stage (Supplementary Table S2). Higher risk scores for the ieILC1/NK clusters were significantly correlated with shorter OS and PFS (Figure 4D). Subsequently, multivariate regression analysis was performed to further evaluate the prognostic significance of the ieILC/NK clusters (Table 1). The T factor, M factor, and ieILC1-1, ieILC1–NK-int, and NK risk scores were identified as independent prognostic factors for the OS, while the ieILC1-1, ieILC1–NK-int, and NK scores were independent prognostic factors for PFS. Based on the results of the multivariate regression analysis, recursive partitioning was performed to construct a multivariate risk model. Three prognostic factors, ieILC1-1, ieILC1–NK-int, and NK risk scores, were identified for both OS and PFS, resulting in eight terminal nodes in the OS model and 10 terminal nodes in the PFS model (Figure 4E,F,H,I). Conditional inference trees indicated that ieILC1-1 was the top predictor of both OS and PFS. Moreover, patients with high ieILC1-1 and NK risk scores had the shortest OS and PFS. Based on the survival time of each terminal node, we merged the terminal nodes and identified three risk groups for the OS and PFS (Figure 4G,J). The three risk groups stratified the patients based on their survival.

## 4. Discussion

To improve the efficacy of cancer immunotherapy, it is crucial to elucidate the complex network of immune cells in the TME. Similar to T cells, ILCs exhibit both antitumoral and protumoral activities, indicating their heterogeneous populations and various functional states [16,17,18]. In the current study, we identified heterogeneous subsets of group 1 ILCs in patients with HNSCC. Moreover, we developed prognostic prediction models based on DEGs in group 1 ILC subsets. Our results provide new insights into the landscape and prognostic significance of group 1 ILCs in patients with HNSCC.

We analyzed scRNA-seq data of tumor-derived CD45+ cells and peripheral blood cells and identified two subsets of ieICL1, eight NK clusters, and an ieILC1–NK-intermediate cluster. Upon comparing the two ieILC1 subsets, we observed that ieILC1-1 exhibited higher expression levels of *IFNG*, *PDCD1*, *LAG3*, *CTLA4*, and *TIGIT* than ieILC1-2. Moreover, ssGSEA revealed that the pathways related to effector function, cell growth, and cell metabolism were enriched in ieILC1-1 cells. Based on the hypothesis that ILCs are the innate counterparts of T cell immunity, our results indicate that the ieILC1-1 cluster is comparable to T cell exhaustion (Tex) progenitor 2 and Tex intermediate, which are both subsets of Tex [27,28]. Tex populations are heterogeneous, with multiple levels of heterogeneity, including Tex progenitor 1, Tex progenitor 2, Tex intermediate, and terminal exhausted T cell. Among Tex subsets, the Tex progenitor 2 subset exhibits increased proliferative capacity, while the Tex intermediate subset demonstrates increased effector function [27,28]. The increased expression of checkpoint molecules and the enriched pathways in ieILC1-1 suggest their Tex progenitor 2-like and Tex intermediate-like states during the exhaustion process of group 1 ILCs. Notably, Tex progenitor subsets and Tex intermediate subsets are the main recipients of immune checkpoint blockade and relate to immunotherapy response [27]. Together with the elevated expression of checkpoint molecules, ieILC1-1 could be a potential target for future immunotherapy. In addition, most ieILC1s isolated from HPV-positive tumors were ieILC1-1. HPV-positive HNSCCs are highly immunogenic owing to viral oncoproteins E6 and E7 and are characterized as “hot” TME [29,30,31]. Our results suggest that ieILC1-1 infiltration into the TME could be one of the vital components of “hot” TME in HPV-positive HNSCC. Two ieILC1 subsets, ieILC1 and ieILC1-cycling, were reported previously and appear comparable to ieILC1-2 and ieILC1-1, respectively [32]. Our results further elucidate the functional heterogeneity of ieILC1s in terms of the HPV status. Elevated ieILC1-1 (ieILC1-cycling) levels could be among the hallmark features of HPV-positive HNSCC.

The expression of granzymes and *PRF1* was similar in ieILC1s and NK cells, while the expression of *IFNG* in ieILC1s, particularly in ieICL1-1 cells, was higher than in NK cells. Both ieiLC1s and NK cells express Eomes and T-bet and produce IFN-γ in response to IL-12 and IL-15 stimulation [8]. Notably, IFN-γ is a cytokine exerting both antitumoral and protumoral functions [33]. IFN-γ exhibits antitumoral effects by inducing tumor cell apoptosis, polarizing tumor-associated macrophages into an M1-like phenotype, and activating effector immune cells [33,34,35]. Meanwhile, IFN-γ exerts protumoral functions by inducing immune checkpoint receptors, including programmed cell death 1 and indoleamine 2,3-deoxygenase in tumor cells, enhancing the metastatic ability of tumor cells while suppressing the migration of cytotoxic T cells [36,37,38,39,40]. Notably, hallmark pathways linked to effector function were upregulated in ieILC1s compared with NK cells, suggesting that IFN-γ is likely associated with greater antitumoral activity in ieILC1s than in NK cells. Among NK cells, CD57+ NK cells, the most abundant NK cells in our results, are a terminally differentiated subset of NK cells and exhibit a higher cytotoxic capacity than CD57− NK cells [11]. Our results suggest that the differentiation of CD57− NK cells into CD57+ NK cells is possibly elevated in the peripheral blood of patients with HNSCC.

Herein, we developed prognostic prediction models based on the DEGs in each ieILC1/NK cluster. The risk scores of each ieILC1/NK cluster calculated using LASSO regression analysis stratified patients with HNSCC. Moreover, the risk scores of the ieILC1-1, ieILC1–NK-int, and NK clusters were identified as independent prognostic factors for shorter OS and PFS. Accumulating evidence indicates that NK cells correlate with a better prognosis in patients with solid tumors, including hepatocellular carcinoma, squamous cell lung cancer, and HNSCC [41,42,43]. However, our results contradict the previously reported findings. In previous studies, NK cells were defined based on the expression of limited surface markers such as CD56 and CD57. The definition of NK cells as CD56+ cells or CD57+ cells could include other immune cell subsets, especially ILC1s. In the current study, we constructed risk scores based on the DEGs of NK cells that significantly correlated with prognosis in univariate Cox regression analysis. Our results suggest that broader recognition of cell populations based on gene expression profiles would enable better prognostic prediction. Moreover, our results demonstrated that both ieILC1-1 and ieILC1–NK-int are independent prognostic factors for shorter survival. Nevertheless, the prognostic significance of ILC1s remains controversial [24,44,45]. The presence of poorly cytotoxic IFN-γ^low^ ILC1s was shown to correlate with an unfavorable prognosis in several cancers. In the current study, ieILC1-1 exhibited a higher *IFNG* expression but was associated with shorter survival. This discrepancy may be attributed to several factors, including the dual-natured functions of IFN-γ in the TME, exhaustion-like phenotype of ieILC1s, and broader definition of clusters based on gene expression profiles. We further developed a prognostic prediction model using recursive partitioning, resulting in clear patient stratification. Particularly, the combination of ieILC1-1 and NK clusters predicted patients with the shortest OS and PFS. Our results demonstrate the possibility of using group 1 ILCs as prognostic markers for patients with HNSCC.

A limitation of the present study is that all results were generated using a publicly available database, necessitating further verification using in vitro, in vivo, and ex vivo experiments.

## 5. Conclusions

The present study demonstrates the diverse landscape and prognostic significance of group 1 ILCs in patients with HNSCC. Further investigation of group 1 ILCs will provide new insights into the development of cancer immunotherapies and biomarkers for patients with HNSCC.

## Figures and Tables

**Table 1:** Univariate and multivariate survival analyses of the OS and PFS in 520 patients with HNSCC.

Variables	Overall Survival			Progression-Free Survival		
	Univariate	Multivariate		Univariate	Multivariate	
	*p*-Value	HR (95% CI)	*p*-Value	*p*-Value	HR (95% CI)	*p*-Value
HPV status (ref.: negative)						
Positive	0.137			0.05		
Primary lesion (ref.: hypopharynx)						
Larynx	0.277			0.095		
Oral cavity	0.395			0.215		
Oropharynx	0.058			0.057		
T factor (ref.: T1–2)						
T3–4	0.0002	1.907 (1.154–3.151)	0.012	0.001		0.206
N factor (ref.: negative)						
Positive	0.037	1.410 (0.967–2.056)	0.074	0.063		
M factor (ref.: M0)						
M1	0.003	4.588 (1.606–13.108)	0.004	0.284		
TNM stage (ref.: I–II)						
III–IV	0.01	0.762 (0.388–1.497)	0.430	0.011	1.554 (0.816–2.961)	0.180
ieILC1-1 risk score (ref.: low)						
High	<0.0001	1.615 (1.041–2.506)	0.033	<0.0001	1.602 (1.017–2.524)	0.042
ieILC1-2 risk score (ref.: low)						
High	<0.0001	1.598 (0.996–2.563)	0.052	<0.0001	1.179 (0.747–1.862)	0.479
ieILC1–NK-int risk score (ref.: low)						
High	<0.0001	1.663 (1.097–2.520)	0.017	<0.0001	2.102 (1.363–3.243)	0.0007
NK risk score (ref.: low)						
High	<0.0001	1.689 (1.173–2.432)	0.005	<0.0001	1.726 (1.134–2.627)	0.011

Note: PFS, progression-free survival; OS, overall survival; HNSCC, head and neck squamous cell carcinoma; HPV, human papillomavirus; HR, hazard ratio; CI, confidence interval.

## Data Availability

The original data presented in this study are openly available in GSE164690, https://www.ncbi.nlm.nih.gov/geo/query/acc.cgi?acc=GSE164690 (accessed on 1 June 2023); GSE112813, https://www.ncbi.nlm.nih.gov/geo/query/acc.cgi?acc=GSE112813 (accessed on 13 August 2023); and on the FireBrowse website, http://firebrowse.org/.

## Supplementary Materials

The following supporting information can be downloaded from: https://www.mdpi.com/article/10.3390/cancers17122047/s1, Supplementary Figure S1: Additional data to Figure 1; Supplementary Figure S2: Additional data to Figure 2; Supplementary Figure S3: Additional data to Figure 4; Supplementary Table S1: Results of univariate regression analyses of the overall survival and progression-free survival using a Cox proportional hazards model; Supplementary Table S2: Relationship between risk scores and clinical parameters in 520 patients with HNSCC.
