# Supplementary material for: Diverse Landscape of Group 1 Innate Lymphoid Cells Predicts the Prognosis in Patients with Head and Neck Squamous Cell Carcinoma
Source: Cancers (Basel). 2025 Jun 19;17(12):2047. doi: 10.3390/cancers17122047 (PMC12191157; doi:10.3390/cancers17122047)
Supplement: Supplementary file 1 [file cancers-17-02047-s001.zip › Supplementary Figures.pdf]

Supplementary Figure S1

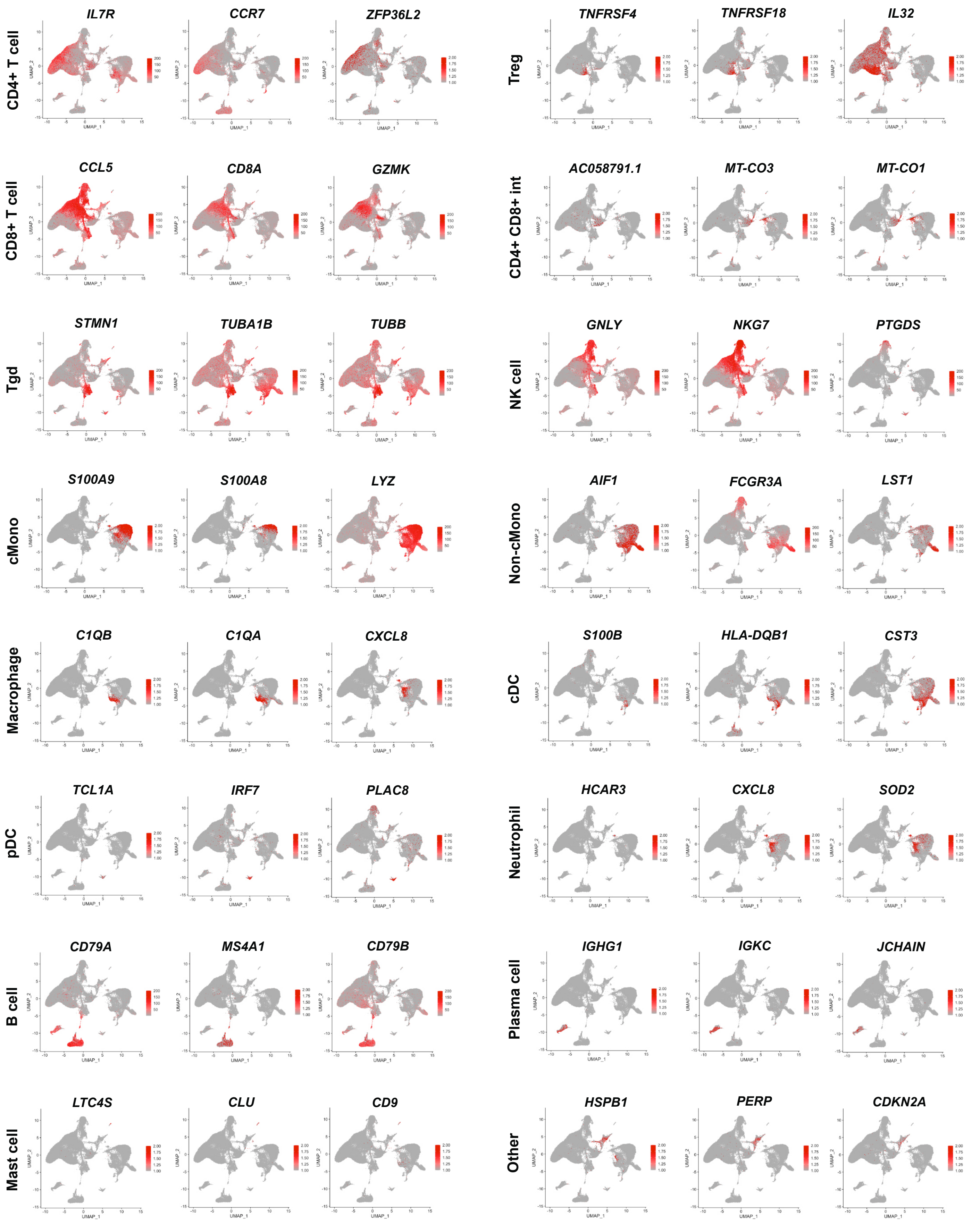

Supplementary Figure S1. Additional data to Figure 1
UMAPs showing the expression of top 3 differentially expressed genes in 16 immune cell types.

Supplementary Figure S2

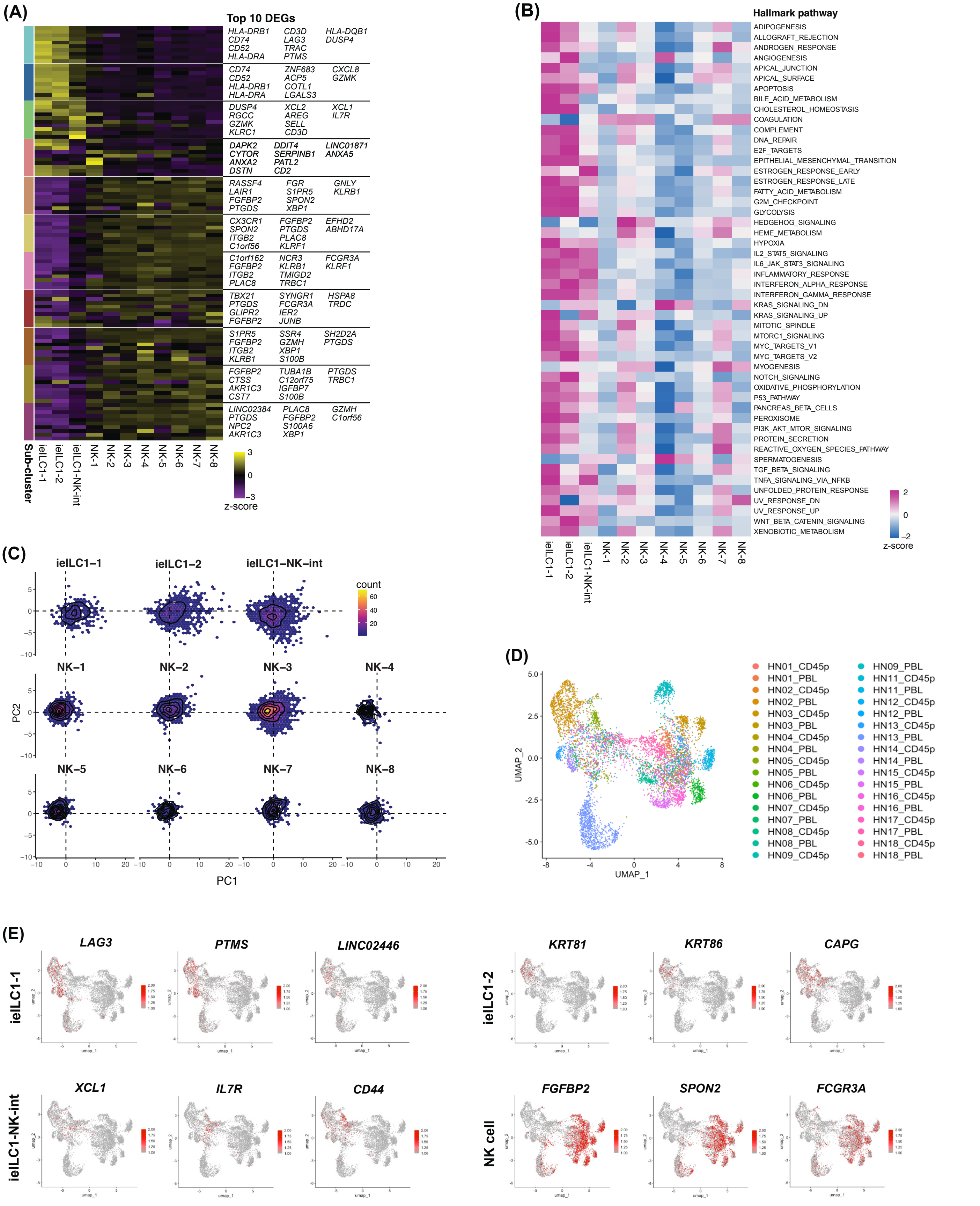

Supplementary Figure S2. Additional data to Figure 2

A, Heat map showing the top DEGs in ieILC1/NK clusters. B, Heat map showing the ssGSEA scores in ieILC1/NK clusters. C, PCA plot based on the ssGSEA scores in ieILC1/NK clusters. D, UMAP showing the distribution of cells based on samples. E, UMAPs showing the expression of top 3 DEGs in ieILC1/NK clusters. UMAP, Uniform Manifold Approximation and Projection; ieILC, intraepithelial innate lymphoid cell. DEG, differentially expressed gene; ssGSEA, single sample gene set enrichment analysis; PCA, principal component analysis.

Supplementary Figure S3

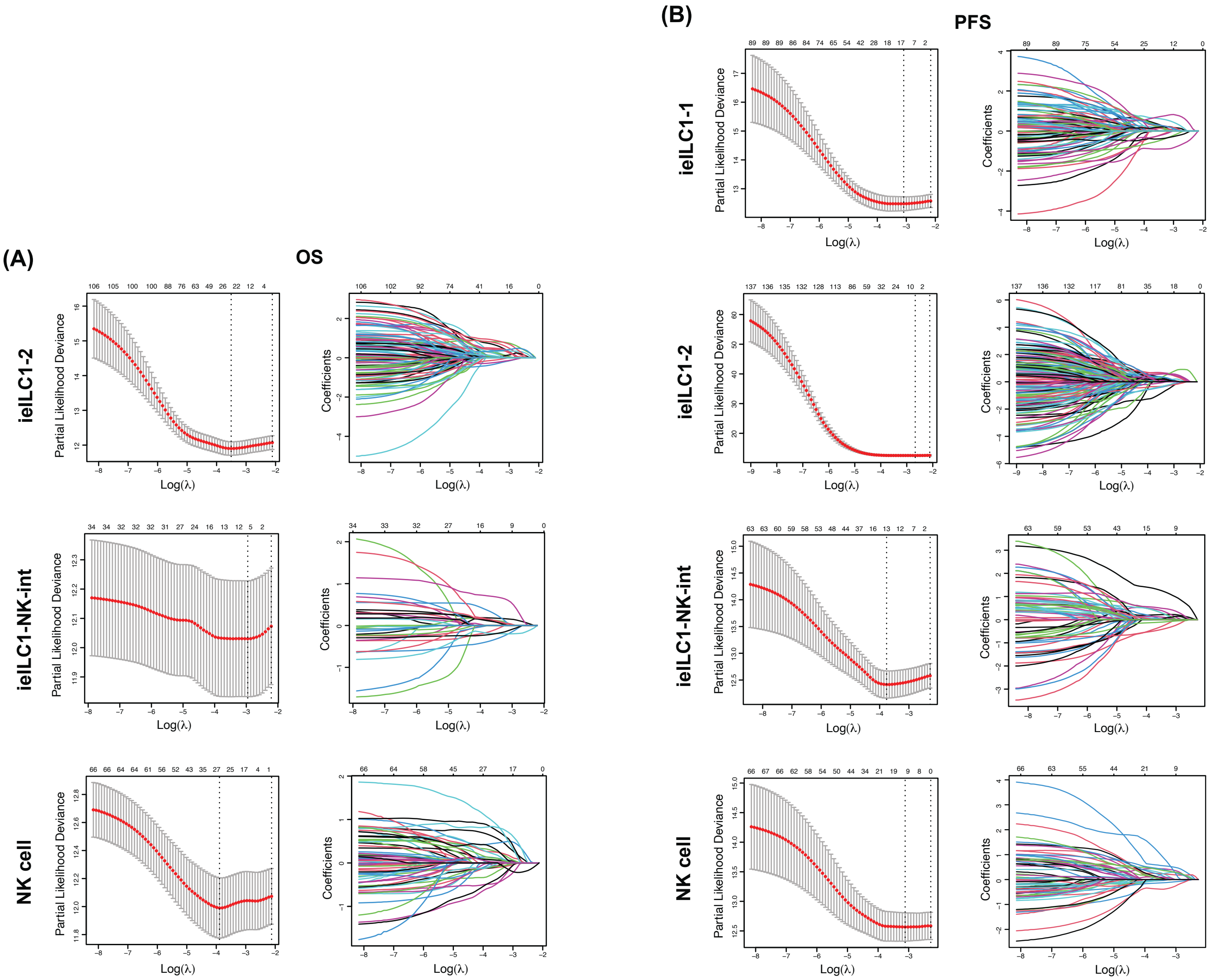

Supplementary Figure S3. Additional data to Figure 4

A, (left) Plot showing partial likelihood deviance versus log (lambda) associated with overall survival. The vertical dotted line indicates the lambda value with the minimum error and the largest lambda value in which deviance was within one standard error of the minimum. (right) LASSO coefficient profiles of DEGs associated with overall survival. B, (left) Plot showing partial likelihood deviance versus log (lambda) associated with progression-free survival. The vertical dotted line indicates the lambda value with the minimum error and the largest lambda value in which deviance was within one standard error of the minimum. (right) LASSO coefficient profiles of DEGs associated with progression-free survival. LASSO, least absolute shrinkage and selection operator; ieILC, intraepithelial innate lymphoid cell; DEG, differentially expressed gene.
